# Invasive Assessment of Hemodynamic, Metabolic and Ionic Consequences During Blood Flow Restriction Training

**DOI:** 10.3389/fphys.2020.617668

**Published:** 2020-12-16

**Authors:** Alexander Franz, Felix Berndt, Joachim Raabe, Jan-Frieder Harmsen, Christoph Zilkens, Michael Behringer

**Affiliations:** ^1^Department of Orthopedics, University Hospital Duesseldorf, Düsseldorf, Germany; ^2^Department of Adult Reconstruction, ATOS Orthoparc Clinic Cologne, Cologne, Germany; ^3^Department of Anesthesiology, University Hospital Duesseldorf, Düsseldorf, Germany; ^4^Department of Nutrition and Movement Sciences, School for Nutrition and Translational Research in Metabolism, Maastricht University Medical Center, Maastricht, Netherlands; ^5^Department of Sports Medicine and Exercise Physiology, Goethe University Frankfurt, Frankfurt, Germany

**Keywords:** venous occlusion, kaatsu training, rehabilitation, hyperkalemia, acidosis, resistance training, physical training

## Abstract

**Purpose:** Medically recommended training often faces the dilemma that necessary mechanical intensities for muscle adaptations exceed patients' physical capacity. In this regard, blood flow restriction (BFR) training is becoming increasingly popular because it enables gains in muscle mass and strength despite using low-mechanical loads combined with external venous occlusion. Since the underlying mechanisms are still unknown, we applied invasive measurements during exercise with and without BFR to promote physiological understanding and safety of this popular training technique.

**Methods:** In a randomized cross-over design, ten healthy men (28.1 ± 6.5 years) underwent two trials of unilateral biceps curls either with (BFR) and without BFR (CON). For analysis of changes in intravascular pressures, blood gases, oximetry and electrolytes, an arterial and a venous catheter were placed at the exercising arm before exercise. Arterial and venous blood gases and intravascular pressures were analyzed before, during and 5 min after exercise.

**Results:** Intravascular pressures in the arterial and venous system were more increased during exercise with BFR compared to CON (*p* < 0.001). Furthermore, arterial and venous blood gas analyses revealed a BFR-induced metabolic acidosis (*p* < 0.05) with increased lactate production (*p* < 0.05) and associated elevations in [K^+^], [Ca^2+^] and [Na^+^] (*p* < 0.001).

**Conclusion:** The present study describes for the first time the local physiological changes during BFR training. While BFR causes greater hypertension in the arterial and venous system of the exercising extremity, observed electrolyte shifts corroborate a local metabolic acidosis with concurrent rises in [K^+^] and [Na^+^]. Although BFR could be a promising new training concept for medical application, its execution is associated with comprehensive physiological challenges.

## Introduction

Training patients to maintain or improve muscle mass often faces a dilemma as the lower limit of recommended loads for resistance training (>50% one repetition maximum, 1RM) often exceeds the patients' physical capacity. Over the last two decades, however, there has been growing evidence that the original recommendations for exercise intensity are no longer tenable. According to these data, 20–30% of the individual 1RM is sufficient to achieve muscle mass and strength gains when the blood supply to the working muscles is restricted and venous return is blocked (Patterson et al., 2019). This type of training is known as blood flow restriction training (BFR-training) and is becoming increasingly popular in recreational and competitive sports. However, due to the low mechanical load required, this training method has recently moved into the clinical focus (Cristina-Oliveira et al., 2020).

Numerous studies are already available that have examined BFR-training in the context of rehabilitation and prehabilitation of different entities (Hughes et al., 2019; Lu et al., 2020). Especially in orthopedic rehabilitation, BFR-training is expected to play a major role in the future since its application can reduce postoperative muscle loss and arthrogenic muscle inhibition (Hughes et al., 2017; Conceição and Ugrinowitsch, 2019). However, most available studies focus on improving physical fitness, leaving a lack of knowledge about the training-related changes in the occluded extremity. Venous pooling raises questions like what pressures the venous vascular system is exposed to and what electrolyte changes occur in the unilaterally closed compartment. Therefore, it can currently only be speculated to which physiological stress the body is exposed during BFR-training (Minniti et al., 2020). To assess the physiological consequences of BFR-training on a local and systemic level, we performed an invasive catheter approach to measure changes in hemodynamic, metabolic and ionic balance during low-load BFR-training in both the arterial and venous system.

## Materials and Methods

### Subjects

Ten healthy male subjects (age: 28.1 ± 6.5 years) volunteered for this study (Table 1). All subjects were experienced in resistance training, had no prior experiences with BFR-Training and reported not having performed regular strength training 1 week before the start of the study. Subjects were informed about the experimental procedures and possible risks and signed an informed consent document before the investigation. The study was approved by the local Ethics Committee of the University Hospital Duesseldorf (Trial-ID: 2015104498) and was performed according to the Declaration of Helsinki.

**Table 1:** Basic characteristics of subjects.

**Subjects (*****n*** **=** **10)**	
**Height (cm)**	**185.6 ± 8.9 cm**
**Weigth (kg)**	**86.7 ± 8.0 kg**
**1RM/30% of 1RM**	**23.4 ± 3.4 kg/7.0 ± 1.0 kg**
**LOP/50% of LOP**	**156 ± 13 mmHg/77.9 ± 6.5 mmHg**

### Study Design

To investigate the effects of BFR on local and systemic physiological adaptations in comparison to a training without BFR, a randomized cross-over design was applied. The ten included subjects performed an exercise protocol for the elbow flexors for two times, once with and once without BFR, separated by 4 weeks of rest. To control the impact of the repeated-bout-effect (Hyldahl et al., 2017), participants were randomized via a random-number table to start with either the control training trial (CON) (*n* = 5) or the BFR training session (BFR) (*n* = 5). Therefore, all subjects reported to the laboratory for three testing sessions and follow-up measurements.

**Figure 1:**
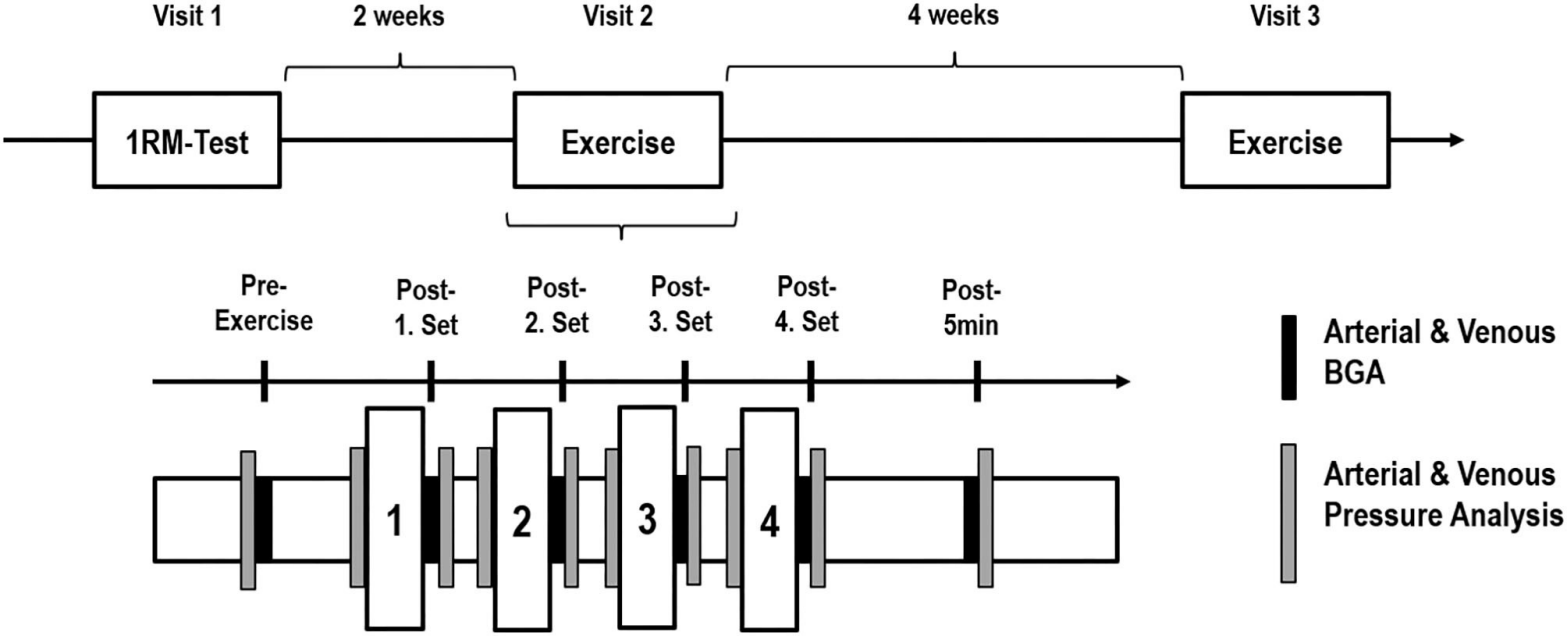
Study design. **(Above)** Graphical overview of the three appointments on a timeline with the corresponding rest phases. **(Below)** Illustration of the experimental protocol with four sets and the time-points of sample and data collection. 1RM, one repetition maximum; BGA, blood gas analysis.

During the first visit, subjects' individual concentric 1RM of the elbow flexor of the dominant arm was determined in accordance to Jessee et al. (2017). After 2 weeks of rest, the second visit was performed by carrying out the experimental loading for the first time. After 4 weeks, the third laboratory visit was performed to repeat the experimental loading as cross-over (Figure 1).

### Sample Size Calculation

Although to the best of our knowledge no data are available on the change in intravenous pressure during BFR training, we assumed that venous pooling leads to a moderate to high effect (*f* = 0.3) with respect to this main outcome. Based on this assumption, we calculated for a repeated measures design (within-between interaction) a required sample size of 10 subjects using the G^*^Power software version 3.1.9.4. The minimal significance (α) and statistical power (1–β) were set at 0.05 and 0.80, respectively.

### Interventions

The exercise protocol consisted of unilateral biceps curls with the dominant arm by using a dumbbell (ScSports, Emmerich, Germany) that were performed for four sets, containing 75 repetitions (reps) (1. Set: 30 reps, 2.-4. Set: 15 reps), at 30% of subjects individual concentric 1RM. The rest between sets was 30 s. Subjects were standing in an upright position, with the back leaning against a wall. Throughout the execution of the exercise, the elbow continuously held contact with the wall. For each repetition, the dumbbell was lifted to a full elbow flexion (~ 50 degrees). The duration of each repetition was set to 4 s, 2 s for the concentric as well as for the eccentric phase, controlled by a metronome (60 beats per min). The set was stopped when volutional failure occured or if the subject was not able to keep the pace.

The BFR-intervention was performed by using 50% of the individual arterial limb occlusion pressure (LOP). For determination of the LOP, the inflatable tourniquets of 11.5 cm width were placed proximal at the exercising arm before the training session (PBFR, Delfi medical Inc., Vancouver, Canada). After a 10-min rest period, LOP was determined sonographically in a lying position by displaying the radial artery with an ultrasound device and using a Doppler to assess the blood flow within the vessel. Subsequently, the cuff was inflated until no further blood flow was detectable. This pressure was defined as the individual LOP.

### Arterial and Venous Blood Sampling and Analysis

**Figure 2:**
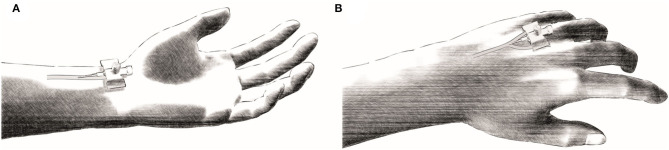
Schematic representation of the puncture locations for the invasive catheters. **(A)** Under local anesthesia the radial artery was punctured sonographically for the placement of the arterial catheter. **(B)** The venous catheter was placed in the venous plexus (*Rete venosum dorsale manus*) of the back of the hand.

To assess intravascular changes in pressure, blood gases and ionic homeostasis, an arterial as well as a venous catheter were placed into the dominant arm before exercise (Zavorsky et al., 2005). For this purpose, subjects were placed in a lying position on a standard medical examination table. Under local anesthesia with lidocaine hydrochloride, Seldinger's technique was used to puncture the radial artery for the arterial access and a dorsal hand vein (*Rete venosum dorsale manus*) for the venous access, respectively (Figure 2).

For determination of intravascular pressures, sensor needles (20 gauge) linked to a line containing a continuous column of saline connected to a transducer unit were used (LogiCal Pressure Monitoring Kit, Smiths medical int. Ltd., UK). The system was calibrated outside the tissue at the patient's heart level in a standing position. Changes in intravascular pressures, arterial and venous blood gas analyses (BGA) (safePICO sampler, Radiometer GmbH, Krefeld, Germany) were collected before exercise, in between the four exercise sets as well as immediately- and 5 min after the exercise protocol (Figure 1). Prior to each collection of samples for BGA, an amount of about 5 ml blood was initially collected from the catheters with a syringe and discarded. Subsequently, a total volume of about 2 ml was taken for each BGA at each time point from the arterial and venous catheter. Since we measured arterial and venous BGA at six time points, a total volume of about 84 ml was taken throughout one test session. The obtained BGA were analyzed directly via the ABL800 FLEX BGA system (Radiometer GmbH, Krefeld, Germany). All examinations, preparations and maintenance associated with the invasive work on the catheters were carried out under sterile conditions.

For monitoring of cardiovascular responses, systolic blood pressure (SBP), diastolic blood pressure (DBP), mean arterial pressure (MAP) and peripheral venous pressure (PVP) were determined. Additionally, the following BGA-parameters were obtained from the arterial (a-) and venous (v-) system: Carbon dioxide partial pressure [p(CO_2_)], oxygen partial pressure [p(O_2_)], oxygen content (ctO_2_), oxyhemoglobin fraction (FO_2_Hb), hemoglobin content (ctHb), oxygen half-saturation pressure of hemoglobin (p50) as well as the concentration of lactate [(La^−^)], potassium [(K^+^)], sodium [(Na^+^)], calcium [(Ca^2+^)], bicarbonate [(${\text{HCO}}_{3}^{-}$)], and chloride [(Cl^−^)].

### Statistics

Two-way repeated measures ANOVAs were used to compare changes in measures over time between BFR and CON. In cases of no condition by time interaction, we interpreted the main effects for condition (BFR vs. CON) and time, and in the case of statistical significance, we followed up the respective main effects with pairwise comparisons using Bonferroni *post hoc*-tests. By contrast, in cases of a significant condition by time interaction, we tested for simple main effects. All statistical analyses were performed with the GraphPad Prism 8 software package (GraphPad Software, San Diego, CA, USA). Results are expressed as means ± standard deviations. Statistical significance was accepted at *p* < 0.05.

## Results

In the BFR trial, participants were not able to perform the same amount of repetitions beginning in the second set leading to a shorter time under tension in the last three sets (*p* < 0.001). Table 2 presents outcomes related to exercise performance during the four sets. At Pre, before exercise, no significant differences between conditions were detected in any outcome parameter.

**Table 2:** Outcomes related to exercise performance during the four sets with blood flow restriction (BFR) and without (Control).

						***p*****-values from 2 way ANOVA**		
	**Condition**	**Set 1**	**Set 2**	**Set 3**	**Set 4**	**Time**	**Condition**	**Interaction**
Repetitions [*n*]	Control	30 ± 0	15 ± 0	15 ± 0	15 ± 0	<0.001	<0.001	<0.001
	BFR	29 ± 2	11 ± 3^#^	8 ± 2^#^	7 ± 2^#^			
Time under tension [s]	Control	113 ± 6	58 ± 5	58 ± 6	57 ± 6	<0.001	<0.001	<0.001
	BFR	114 ± 7	50 ± 6^#^	40 ± 8^#^	35 ± 8^#^			
Rest period after set [s]	Control	37 ± 6	37 ± 5	36 ± 5	n.a.	0.287	0.921	0.776
	BFR	38 ± 7	37 ± 10	35 ± 6	n.a.			

# *Significantly different between conditions (p < 0.05)*.

### Cardiovascular Outcomes

**Figure 3:**
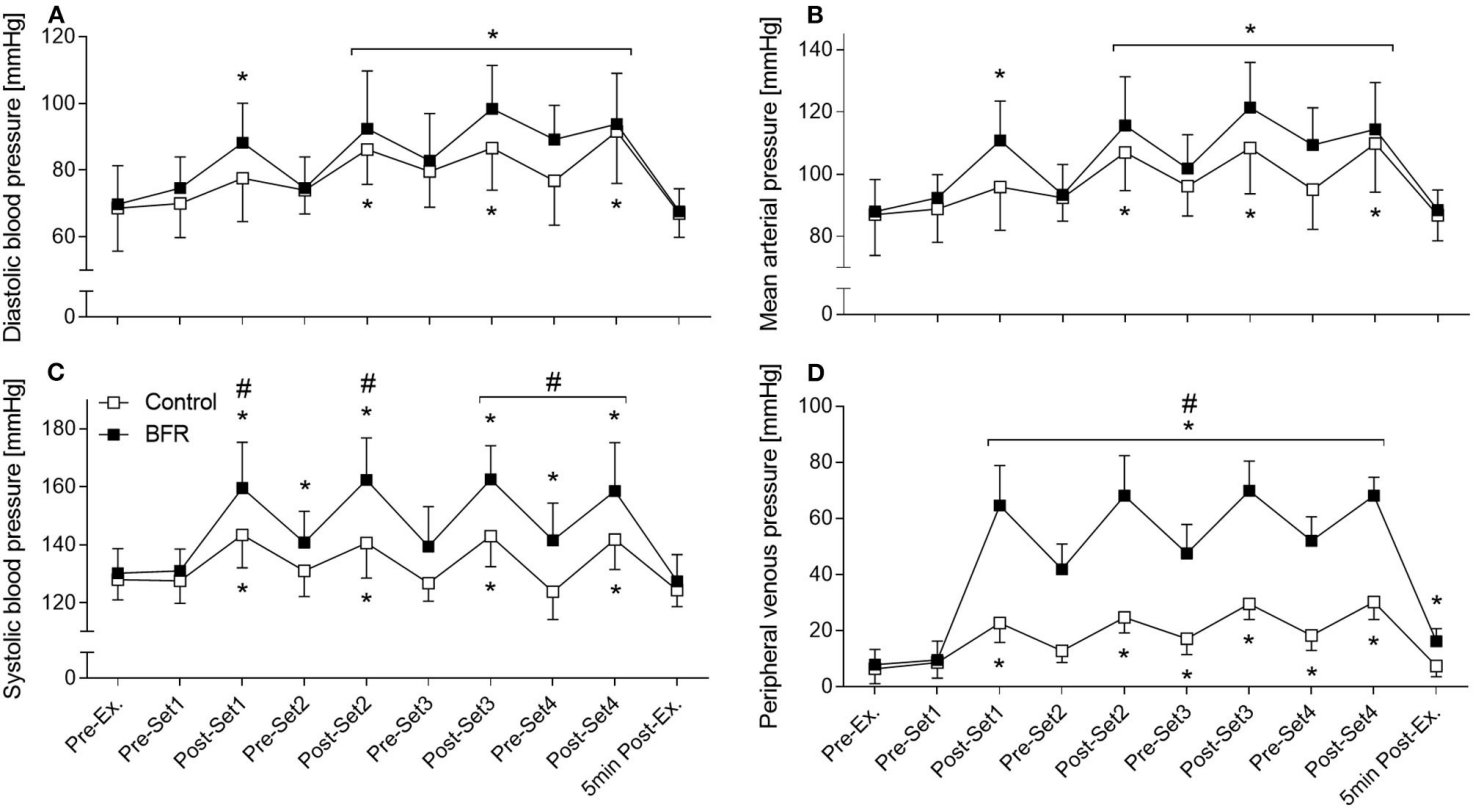
Intravascular pressure parameters. **(A)** diastolic blood pressure, **(B)** mean arterial pressure, **(C)** systolic blood pressure, **(D)** peripheral venous pressure. *Significantly different from Pre within the respective condition (*p* < 0.05). ^#^Significantly different between conditions (*p* < 0.05).

Heart rate (HR) and all intravascular pressure parameters increased showing significant time effects (*p* < 0.001) as shown in Figure 3. For HR, DBP (Figure 3A), and MAP (Figure 3B), there were no significant differences between conditions at any time point. SBP and PVP showed additionally a significant condition (*p* = 0.002 and *p* < 0.001, respectively) and interaction effect (for both *p* < 0.001). SBP was higher in BFR compared to CON after all four sets and at Pre-Set 4 (*p* < 0.05) with the greatest difference at Post-Set 2 (162 ± 15 mmHG vs. 141 ± 12 mmHG, *p* < 0.001, Figure 3C). From Post-Set 1 to Post-Set 4, BFR demonstrated higher PVP values compared to CON (*p* < 0.001) with the greatest difference at Post-Set 1 (65 ± 14 mmHG vs. 23 ± 7 mmHG, Figure 3D).

### Blood Gas Analysis

**Figure 4:**
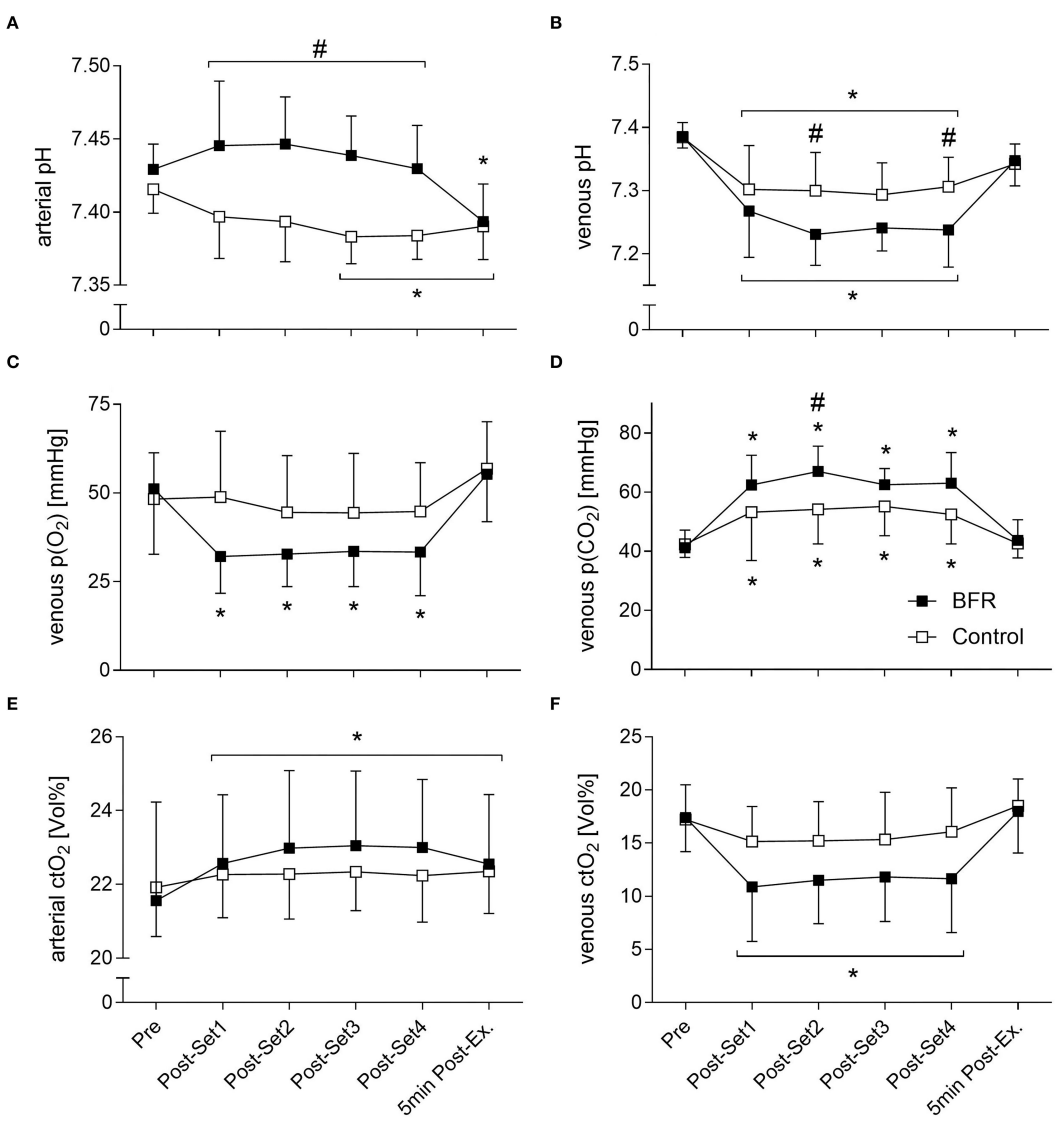
Main blood gas parameters with a significant interaction effect. **(A)** arterial pH, **(B)** venous pH, **(C)** venous p(O_2_), **(D)** venous p(CO_2_), **(E)** arterial ctO_2_, **(F)** venous ctO_2_. *Significantly different from Pre within the respective condition (*p* < 0.05). ^#^Significantly different between conditions (*p* < 0.05).

**Figure 5:**
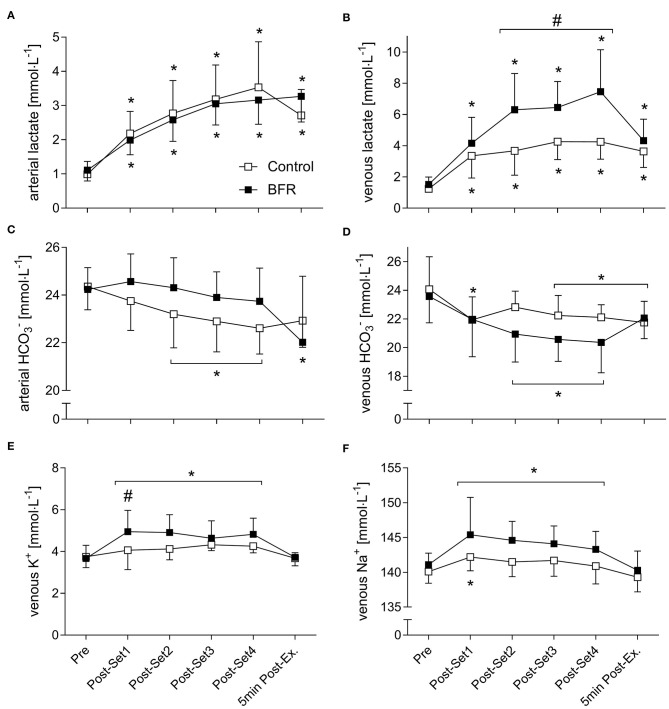
Electrolytes with a significant interaction effect. **(A)** arterial lactate, **(B)** venous lactate, **(C)** arterial bicarbonate (${\text{HCO}}_{3}^{-}$), **(D)** venous ${\text{HCO}}_{3}^{-}$, **(E)** venous potassium (K^+^), **(F)** venous sodium (Na+). *Significantly different from Pre within the respective condition (*p* < 0.05). ^#^Significantly different between conditions (*p* < 0.05).

Arterial and venous pH, p(O_2_), p(CO_2_), p50, ctO_2_, ctHb, [K^+^], [Na^+^], [La^−^], [${\text{HCO}}_{3}^{-}$], and venous FO_2_Hb all showed significant time effects. A significant condition effect was revealed for arterial and venous p(CO_2_) and pH, and venous ctHb, FO_2_Hb, [Na^+^], and [La^−^]. A significant interaction effect was revealed for arterial and venous pH, ctO_2_, [La^−^] and [${\text{HCO}}_{3}^{-}$], and venous p(O_2_), p(CO_2_), p50, FO_2_Hb, [K^+^], [Na^+^] and a-ctHb (for the main blood gases see Figure 4, for electrolytes see Figure 5). All results of outcomes from blood gas analysis that are not presented in figures are summarized in Table 3.

**Table 3:** Additional outcomes related to blood gas analysis including oximetry and electrolyte data.

								***p*****-values from 2 way ANOVA**		
	**Condition**	**Pre**	**Post-Set 1**	**Post-Set 2**	**Post-Set 3**	**Post-Set 4**	**5 min Post-Ex**.	**Time**	**Condition**	**Interaction**
**BLOOD GASES/OXIMETRY**										
a-p(O_2_) [mmHg]	Control	95 ± 5	97 ± 9	102 ± 10	100 ± 10	103 ± 13	102 ± 9	0.015	0.091	0.369
	BFR	97 ± 8	109 ± 13	109 ± 10	106 ± 8	102 ± 8	105 ± 16			
a-p(CO_2_) [mmHg]	Control	38 ± 3	39 ± 4	38 ± 4	38 ± 3	38 ± 3	38 ± 3	0.7912	0.017	0.785
	BFR	36 ± 3	34 ± 6	33 ± 4	33 ± 4	34 ± 5	33 ± 9			
a-p50 [mmHg]	Control	25.8 ± 1.1	26.2 ± 0.9	26.3 ± 0.9	26.8 ± 0.3	26.9 ± 0.5	26.8 ± 0.6	0.009	0.105	0.133
	BFR	25.7 ± 0.5	25.6 ± 2.1	25.6 ± 1.9	25.7 ± 1.5	25.6 ± 0.9	26.7 ± 1.4			
v-p50 [mmHg]	Control	27.1 ± 1.5	30.2 ± 3.6	30.3 ± 2.3	30.7 ± 2.0	30.2 ± 2.3	28.4 ± 1.5	<0.001	0.065	0.029
	BFR	27.3 ± 1.7	32.3 ± 2.6	33.0 ± 2.1	32.6 ± 1.8	32.2 ± 2.3	28.3 ± 2.0			
a.ctHb [g·dL^−1^]	Control	16.0 ± 1.0	16.3 ± 0.9	16.3 ± 0.9	16.3 ± 0.7	16.2 ± 0.9	16.2 ± 0.8	<0.001	0.714	0.039
	BFR	15.7 ± 1.9	16.3 ± 1.3	16.6 ± 1.5	16.7 ± 1.3	16.7 ± 1.3	16.4 ± 1.3			
v.ctHb [g·dL^−1^]	Control	15.6 ± 1.3	15.4 ± 1.9	16.1 ± 0.8	16.6 ± 0.7	16.4 ± 0.7	15.7 ± 1.1	<0.001	0.045	0.239
	BFR	15.6 ± 2.2	16.6 ± 1.5	17.5 ± 0.6	17.5 ± 1.1	17.5 ± 1.4	15.5 ± 1.8			
a.FO_2_Hb [%]	Control	97.2 ± 0.7	97.2 ± 0.8	97.6 ± 0.6	97.4 ± 0.4	97.5 ± 0.6	97.6 ± 0.6	0.096	0.174	0.113
	BFR	97.5 ± 0.6	97.9 ± 0.9	98 ± 0.7	97.9 ± 1.0	97.8 ± 0.7	97.5 ± 0.5			
v.FO_2_Hb [%]	Control	78.7 ± 11.2	71.4 ± 18.6	67.7 ± 17.6	66.2 ± 18.9	69.9 ± 17.9	84.0 ± 8.2	<0.001	0.033	0.002
	BFR	76.7 ± 14.4	46.1 ± 21.5^#^	47.6 ± 18.5	49.2 ± 19.7	48.4 ± 22.8^#^	82.1 ± 13.3			
**ELECTROLYTES**										
a[K^+^] [mmol·L^−1^]	Control	4.0 ± 0.3	4.4 ± 0.2	4.3 ± 0.3	4.4 ± 0.2	4.3 ± 0.4	4.0 ± 0.3	<0.001	0.478	0.210
	BFR	3.8 ± 0.4	4.3 ± 0.3	4.4 ± 0.3	4.3 ± 0.3	4.3 ± 0.4	3.8 ± 0.2			
a[Na^+^] [mmol·L^−1^]	Control	139.4 ± 1.8	139.7 ± 1.9	140.0 ± 2.5	139.3 ± 1.8	138.8 ± 2.2	137.7 ± 1.8	<0.001	0.532	0.205
	BFR	139.1 ± 2.1	138.8 ± 2.0	138.8 ± 1.6	138.7 ± 1.7	138.7 ± 1.9	138.0 ± 0.8			
a[Ca^2+^] [mmol·L^−1^]	Control	1.21 ± 0.03	1.22 ± 0.05	1.22 ± 0.05	1.23 ± 0.04	1.22 ± 0.04	1.21 ± 0.04	0.217	0.334	0.194
	BFR	1.19 ± 0.05	1.21 ± 0.05	1.20 ± 0.04	1.21 ± 0.04	1.21 ± 0.04	1.20 ± 0.04			
v[Ca^2+^] [mmol·L^−1^]	Control	1.18 ± 0.10	1.21 ± 0.14	1.24 ± 0.07	1.26 ± 0.05	1.24 ± 0.06	1.19 ± 0.08	<0.001	0.052	0.862
	BFR	1.18 ± 0.11	1.30 ± 0.11	1.33 ± 0.06	1.29 ± 0.09	1.30 ± 0.07	1.20 ± 0.05			
a[Cl^−^] [mmol·L^−1^]	Control	107.8 ± 2.8	108.7 ± 1.9	109.0 ± 1.8	109.2 ± 2.3	109.0 ± 2.1	108.8 ± 2.2	0.023	0.622	0.235
	BFR	108.3 ± 2.0	108.0 ± 2.2	109.1 ± 2.0	109.4 ± 1.8	110.7 ± 5.2	109.9 ± 4.0			
v[Cl^−^] [mmol·L^−1^]	Control	109.2 ± 4.6	110.2 ± 5.3	109.1 ± 2.6	107.8 ± 2.2	108.9 ± 2.5	110.1 ± 6.1	0.314	0.547	0.383
	BFR	110.7 ± 5.2	108.9 ± 3.9	107.7 ± 2.3	108.3 ± 2.5	107.3 ± 2.2	109.0 ± 2.6			

# *Significantly different between conditions (p <0.05)*.

After each of the four sets, a-pH was higher in BFR compared to CON (*p* ≤ 0.001, Figure 4A). For v-pH, BFR demonstrated a more pronounced drop with lower values at Post-Set 2 (7.23 ± 0.05 vs. 7.30 ± 0.06, *p* = 0.014) and Post-Set 4 (7.24 ± 0.06 vs. 7.31 ± 0.05, *p* = 0.015) compared to CON (Figure 4B). Only in BFR, the v-p(O_2_) was decreased after all four sets compared to Pre (*p* < 0.001, Figure 4C). BFR demonstrated a greater increase in v-p(CO_2_) with higher levels at Post-Set 2 compared to CON (67.0 ± 8.6 mmHG vs. 54.2 ± 11.7 mmHG, *p* = 0.017, Figure 4D). At Post-Set2, v-p50 was higher in BFR compared to CON (*p* = 0.043). V-FO_2_Hb was lower in BFR compared to CON at Post-Set 1 (46.1 ± 21.4% vs. 71.4 ± 18.6%, *p* = 0.009) and Post-Set 4 (48.4 ± 22.8% vs. 69.9 ± 17.8%, *p* = 0.040).

Only in CON, a-[La^−^] were already decreased 5 min Post-Exercise compared to Post-Set 4 (2.7 ± 0.8 mmol/L, *p* = 0.004, Figure 5A). After the second until the fourth set, v-[La^−^] were significantly higher in BFR compared to CON (*p* < 0.05) with the greatest difference Post-Set 4 (7.5 ± 2.7 mmol/L vs. 4.3 ± 1.1 mmol/L, *p* < 0.001, Figure 5B). Whereas, in BFR a-[${\text{HCO}}_{3}^{-}$] were only significantly decreased 5 min Post-Exercise (22.0 ± 2.8 mmol/L vs. 24.2 ± 0.9 mmol/L, *p* < 0.001), reductions in CON started at Post-Set 2 and persisted thereafter (*p* < 0.05, Figure 5C). In CON, reduced v-[${\text{HCO}}_{3}^{-}$] were present at all post measurements (*p* = 0.05) except for Post-Set 2 (*p* = 0.214), and in BFR at Post-Set 2 until Post-Set 4 (*p* < 0.001, Figure 5D).

After each of the four sets v-[K^+^] were only significantly elevated in BFR (*p* < 0.001), and at Post-Set 1 this was higher than in CON (5.0 ± 1.0 mmol/L vs. 4.1 ± 0.9 mmol/L, *p* = 0.019, Figure 5E). In CON, v-[Na^+^] were only significantly elevated at Post-Set 1 (142.2 ± 2.0 mmol/L vs. 140.1 ± 1.7 mmol/L, *p* = 0.048), whereas in BFR, elevations were significant after each of the four sets (*p* < 0.05, Figure 5F). Only in BFR, a-ctHb was elevated at all post-measurements (*p* < 0.01). Figures for all other parameters can be found in the Supplementary Figures 1, 2.

## Discussion

The present study is the first to measure changes in intravascular pressures, blood gases, oximetry, and electrolytes during BFR in the occluded limb.

### Intravascular Pressures and Cardiovascular Response

During exercise, mechanical and/or metabolic stress can induce systemic adjustments within the cardiovascular system, which is called exercise pressor reflex (Spranger et al., 2015; Wan et al., 2020). The present findings support the occurrence of the exercise pressor reflex as unilateral biceps curls with 30% of the 1RM resulted in elevations of HR, SBP and DBP compared to baseline even in CON. Furthermore, our data reveal a greater increase in SBP through BFR after all sets in comparison to CON, which is consistent with previous measurements at the contralateral arm (Brandner et al., 2015) or changes induced by BFR of the lower extremity (Poton and Polito, 2015).

While the present findings rely on young and trained subjects, research in older populations indicate that low intensity BFR can cause higher arterial pressures even in comparison to regular high intensity training (Scott et al., 2018). Therefore, it needs to be highlighted that BFR induces a more pronounced cardiovascular response than low intensity training alone, which has to be considered in the practical application of BFR training in different populations.

In line with the obtained findings in the arterial system, the present data also indicate BFR-induced venous hypertension. In relation to previously reported increased membrane permeability (Wernbom et al., 2012) and reductions in plasma volume upon BFR-training (Sato et al., 2005), venous hypertension could explain the muscle swelling often observed after BFR (Freitas et al., 2017). Venous hypertension suggests an increase in the hydrostatic filtration pressure within the capillary bed supporting the frequently discussed contribution of BFR-induced muscle swelling to muscle anabolism (Loenneke et al., 2012).

However, our findings regarding the impact of BFR on the venous system provide important information on the applicability of this training method. Considering that in a standing position at rest gravity causes a hydrostatic venous hypertension of about 35 mmHg at the hand and 90 mmHg above the ankle (Arnoldi, 1965; Tansey et al., 2019), BFR-training can enhance hypertension by ~60 mmHg. Whereas this condition can be tolerated by a healthy venous system with functioning venous valves which prevent a pathological venous reflux (Sarin et al., 1992), patients with venous insufficiency or postoperative lymphedema could experience worsening of their cardiovascular health status through BFR-training.

### Blood Gases and Oximetry

To quantify changes in oxygen supply during exercise, BGA and oximetry data from arterial and venous blood samples were analyzed. Based on previously reported reductions in arterial blood flow (Kilgas et al., 2019) and tissue oxygenation during BFR (Neto et al., 2016), we hypothesized that the arterial and venous p(O_2_) and ct(O_2_) would be reduced during BFR with concurrently rising p(CO_2_) in the venous system.

The present findings prove a rising metabolic demand in the venous part of the working extremity during BFR by reduced v-p(O_2_) and v-ct(O_2_). Furthermore, the detected right shift of the oxygen binding curve, shown by the reduced v-p50, is likely induced by a local metabolic acidosis, since we observed a rise in v-p(CO_2_) as well. Surprisingly, the oxygen supply in the arterial part of the vascular system was increased during BFR. These results are in contrast with the previous mechanistic approach, where a lower arterial blood flow and subsequent insufficient supply of the muscle tissue during BFR was assumed to increase metabolic stress (Downs et al., 2014).

Interestingly, similar changes in BGA can be found in retinal branch vein occlusions, where a mechanical blockage of the venous return caused reduced v-p(O_2_) but simultaneously elevated levels in the arterial branch (Yang et al., 2017). Concerning the origin of this finding, authors discussed that although oxygen is continuously supplied by the arterial system, it may not reach the undersupplied areas due to the blood stasis (Lin et al., 2016). Since the present findings support this idea indirectly through the observed rise in ctHb during BFR, this purely mechanistical approach does not consider potential exercise-induced changes in membrane permeability or oxygen binding capacity. Consequently, this explanation is probably only a partial answer to this unexpected result and should be investigated in future studies.

### Electrolytes

Based on the demonstrated changes in blood gases we assumed that BFR is a more metabolically demanding training regime with greater impact on metabolism and electrolytes. Although the systemic a-[La^−^] showed no differences between CON and BFR, v-[La^−^] were higher during BFR which is in line with previous reports (Poton and Polito, 2016). In combination with the described changes in oxygen content and v-p(CO_2_), the increased La^−^ formation supports the theory that the incoming oxygen does not reach the capillary bed of the working tissue despite increased availability in the arterial system.

In addition to the acid-base balance, BFR-training shows pronounced effects on the electrolyte balance. In line with previous reports (Lindinger, 1995), a periodic shift from increased [K^+^] during exercise toward a rapid decrease to baseline levels after cessation was evident in both conditions. While increases in v-[K^+^] during normal training are continuously provided to systemic circulation, hyperkalemic states were usually only achievable for a short time due to the necessary high-intensities (Vøllestad et al., 1994). Although arterial levels were not more elevated by BFR, v-[K^+^] were higher after the first set of exercise in comparison to CON, which is rapidly washed out into the circulation when the cuff is opened.

In addition to the occlusion-related concentration of electrolytes, the altered acid-base balance with increased [La^−^] and hydrogen ions ([H^+^]) in BFR is responsible for the accumulation of K^+^ during exercise as well. Rising [H^+^] cause an inhibition of K^+^-reuptake and simultaneously displace K^+^ from binding to cellular proteins (Aronson and Giebisch, 2011). While these results are associated with the faster onset of fatigue during BFR (Allen et al., 2008), this disturbed K^+^-handling is of considerable relevance for the clinical application of the training technique.

Previous studies reported a reduction in muscle Na^+^-K^+^-ATPase density in atrophy-affected skeletal muscles in the elderly (Ford et al., 1993), in degenerative joint diseases (Perry et al., 2013), after immobilization (Jebens et al., 1995) or after surgical interventions (Perry et al., 2015). Since these findings indicate a reduced capacity for K^+^-reuptake (Sejersted and Sjøgaard, 2000), all these conditions could facilitate the onset of hyperkalemic periods (>5.2 mmol/L) if BFR-training is applied. Indeed, if large muscle groups are involved, BFR-induced enhancement of muscle fiber recruitment (Yasuda et al., 2014) cause a linear rise in K^+^-release (Medbø and Sejersted, 1990), which may exceed the reuptake capacity of the affected skeletal muscle tissue (Shushakov et al., 2007). Accordingly, particularly during the first sessions with BFR, post-exercise cuff release is able to cause a temporary rise in systemic [K^+^], which could increase the risk of arrhythmias in patients with compromised cardiovascular health (Paterson, 1996; Gupta et al., 2008).

Taken together, our findings illustrate the acute physiological response of a first time BFR-training, which are significantly reduced after repetitive application of BFR. In this context, Christiansen et al. (2019) found a greater improvement in endurance performance after a 6-week interval training with BFR, which was accompanied by a reduced net K^+^-release from thigh muscles. Since such a physiological adaptation was also associated with a reduced La^−^-release (Christiansen et al., 2020), the importance of the acid-base balance for the homeostasis of [K^+^] becomes evident.

## Clinical Considerations

**Figure 6:**
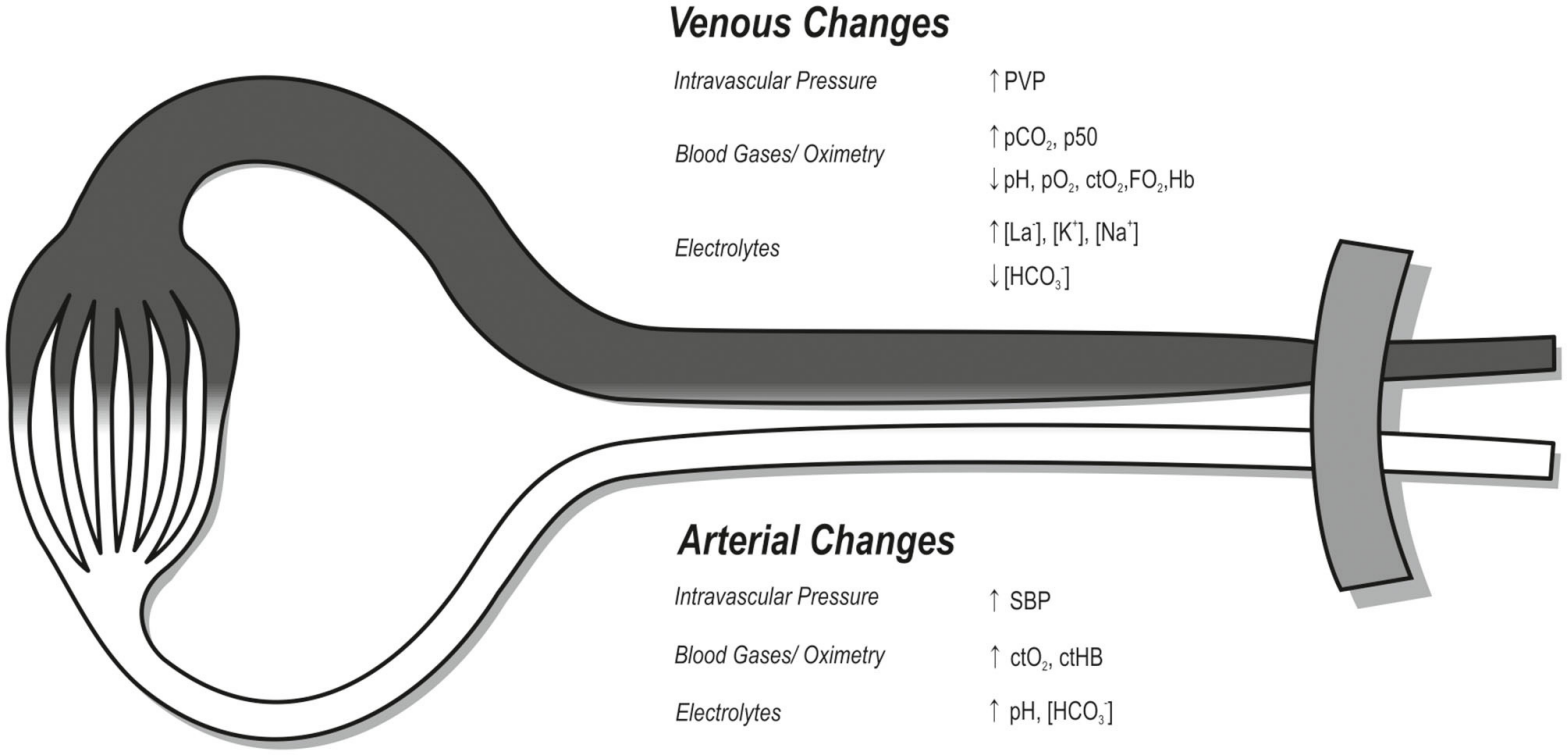
Illustration of significant changes during BFR compared to regular low-intensity training of the upper extremity (*p* < 0.05). While the white area reflects the arterial system, the dark area describes the venous system. The tourniquet commonly used in BFR is located proximally, illustrated in gray. Changes are indicated by arrows for increase (↑) or decrease (↓). SBP, systolic blood pressure; PVP, peripheral venous pressure; pCO_2_, carbon dioxide partial pressure; pO_2_, oxygen partial pressure; ctO_2_, oxygen content; FO_2_Hb, oxyhemoglobin fraction; ctHb, hemoglobin content; p50, oxygen half-saturation pressure of hemoglobin; La^−^, lactate; K^+^, potassium; Na^+^, sodium; ${\text{HCO}}_{3}^{-}$, bicarbonate.

BFR-training provides a new possibility for physically compromised patients to develop or regain muscle strength, mass and prevent atrophy. However, as BFR is associated with higher SBP and PVP, training in patients with vascular disorders might not be appropriate, i.e., hypertension, heart failure or venous insufficiency. The sympathetic overactivity known to be present in these disorders could blunt the metaboreflex-mediated responses required to deal with the increased metabolic demand associated with BFR-training (Spranger et al., 2015). Furthermore, the current knowledge of BFR-associated changes in electrolytes and their impact during exercise and recovery is poor. For this purpose, future studies need to investigate the effects of these acute responses in order to support a safe application in the medical field (Figure 6).

## Limitations

The present study examined the effects of BFR on changes in intravascular pressures, blood gases and electrolytes by using an invasive approach with an arterial and venous catheter system. Although the radial artery originates from the brachial artery which supplies the biceps brachii muscle, its puncture at the wrist and the following analyzation of blood samples do not represent the exact physiological arterial conditions by which the biceps muscle is supplied. Additionally, the *rete venosum dorsale manus* does not reflect the specific drainage area of the biceps muscles, which should be taking into account when interpreting our findings. However, since the invasive catheter method on the upper limbs is routinely not practicable otherwise, we believe that the present findings can be confidently used to interpret changes within the intravascular system during BFR-training.

Furthermore, the sample characteristics as well as the intensity of the compared exercises limits the generalizability of the study outcome. Since the included subjects were experienced in resistance training, former studies in untrained subjects revealed unexpectedly strong physiological responses, even to regular training intensites (Franz et al., 2018). Therefore, the physiological response to BFR-training in a group of untrained subjects would probably be even more pronounced. Additionally, BFR application caused rapid fatigure in the present study so that not all 75 repetitions could be performed, which was different to CON. To assess the valid differences between CON and BFR, future studies should investigate exercise to volutional failure under both conditions.

Finally, from a practical point of view, it should be highlighted that prior to any application of BFR, i.e., in clinical populations, caution should be taken considering that the application of BFR creates a physiological condition in the body that the patient has never experienced before.

## Data Availability Statement

The original contributions presented in the study are included in the article/Supplementary Materials, further inquiries can be directed to the corresponding author/s.

## Ethics Statement

This study was reviewed and approved by the Ethics Committee of the University Hospital Duesseldorf, Moorenstrasse 5, DE-40223, Duesseldorf, Germany. The participants provided their written informed consent to participate in this study.

## Author's Note

The authors affirm that this manuscript is an honest, accurate, and transparent account of the study being reported; that no important aspects of the study have been omitted; and that any discrepancies from the study as planned have been explained.

## Author Contributions

AF was responsible for study conception, conduction, evaluation and manuscript preparation. FB collected and interpretated the non-invasive data and was involved in the realization of the study. JR collected and interpretated the invasive data and was involved in the realization of the study. J-FH was responsible for statistical analyzation of the obtained data and writing the manuscript. CZ and MB were responsible for the conception and preparation of the study protocol, study execution and writing the manuscript. All authors read and approved the final manuscript.

## Conflict of Interest

The authors declare that the research was conducted in the absence of any commercial or financial relationships that could be construed as a potential conflict of interest.

## Supplementary Material

Supplemental Figure 1 Blood gas parameters. ^*^Significantly different from Pre within the respective condition (*p* < 0.05). pO_2_, partial pressure of oxygen; pCO_2_, partial pressure of carbon dioxide; p50, oxygen half-saturation pressure of hemoglobin; ctHb, hemoglobin content; FO_2_Hb, oxyhemoglobin fraction.

Supplemental Figure 2 Electrolytes. ^*^Significantly different from Pre within the respective condition (*p* < 0.05). #Significantly different between conditions (*p* < 0.05). K^+^, potassium; Na^+^, sodium; Ca^2+^, calcium; Cl^−^, chloride.
