# Supplementary figures and images for: Invasive Assessment of Hemodynamic, Metabolic and Ionic Consequences During Blood Flow Restriction Training
Source: Front Physiol. 2020 Dec 16;11:617668. doi: 10.3389/fphys.2020.617668 (PMC7772195; doi:10.3389/fphys.2020.617668)

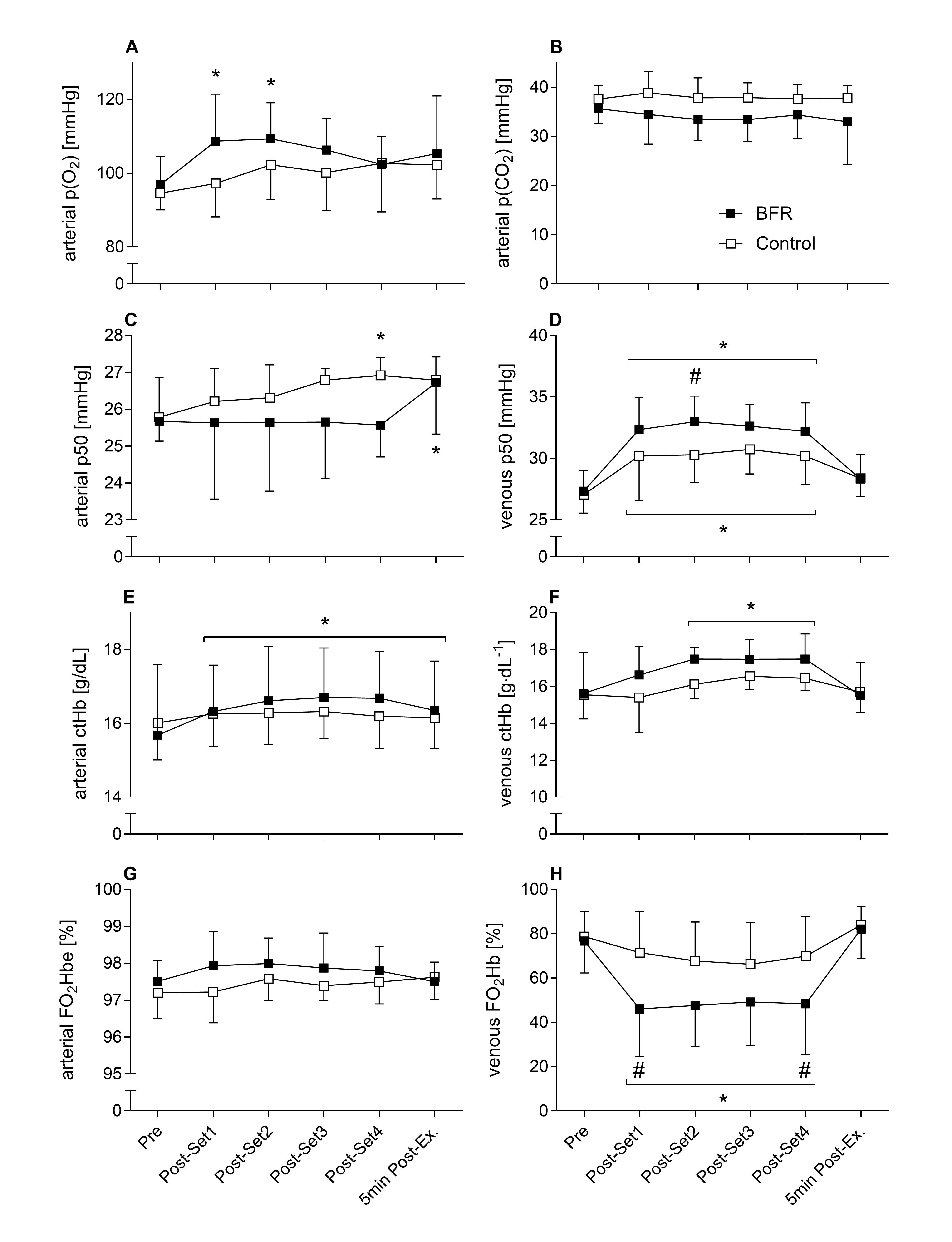

Supplement: Supplemental Figure 1 — Blood gas parameters. *Significantly different from Pre within the respective condition (p < 0.05). pO2, partial pressure of oxygen; pCO2, partial pressure of carbon dioxide; p50, oxygen half-saturation pressure of hemoglobin; ctHb, hemoglobin content; FO2Hb, oxyhemoglobin fraction. [file Image_1.JPEG]

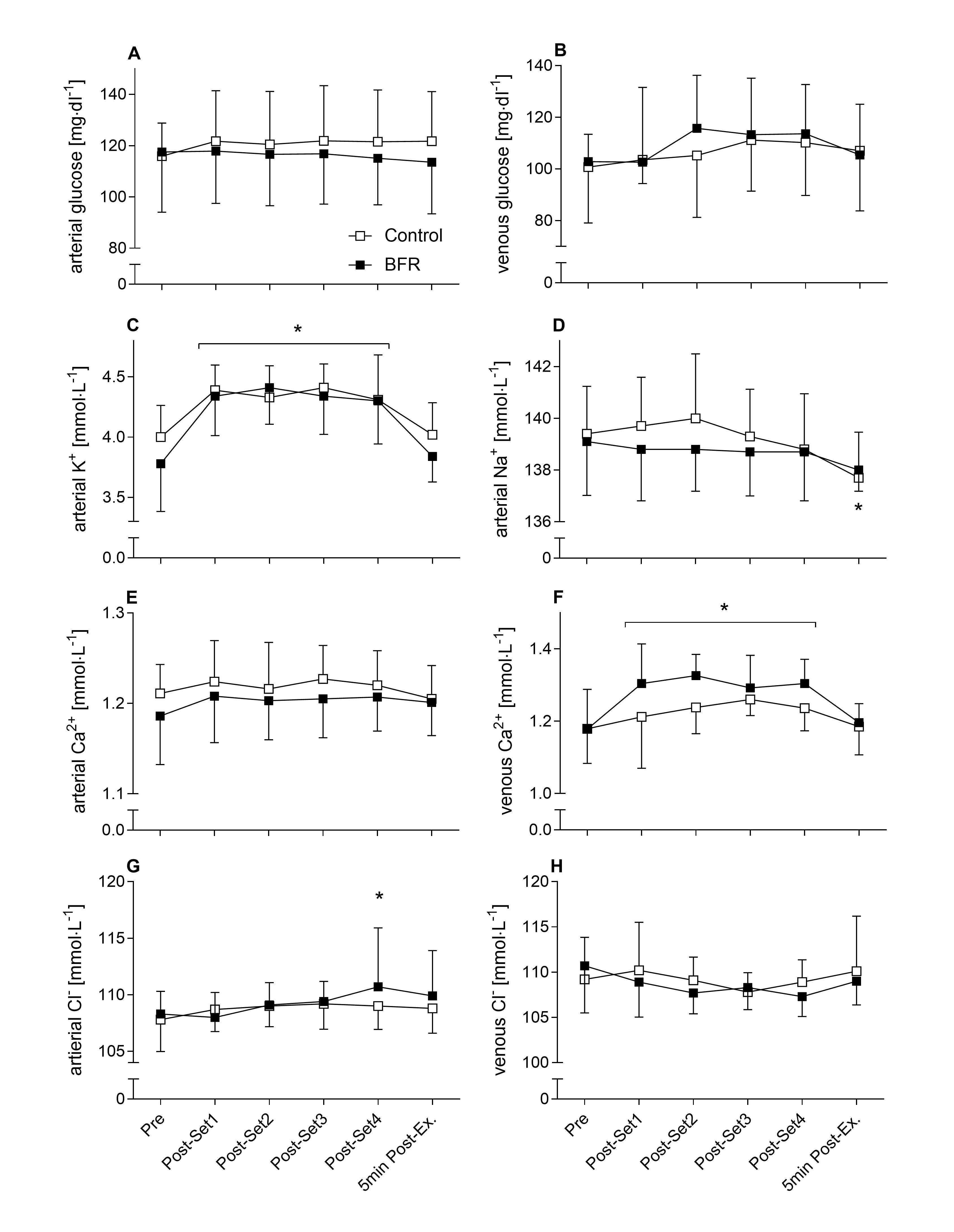

Supplement: Supplemental Figure 2 — Electrolytes. *Significantly different from Pre within the respective condition (p < 0.05). #Significantly different between conditions (p < 0.05). K+, potassium; Na+, sodium; Ca2+, calcium; Cl−, chloride. [file Image_2.JPEG]
